# Post-Traumatic conversion disorder in childhood : A case of functional tetraplegia uncovering cumulative trauma

**DOI:** 10.1192/j.eurpsy.2026.11175

**Published:** 2026-07-31

**Authors:** R. R. Beddiaf, B. Derridj, M. Tabti, Amira Yasmine Benmelouka

**Affiliations:** 1 Medecine, Mahfoud Boucebci Specialized Mental Health Hospital; 2 Faculty of medicine, University of Algiers, Algiers, Algeria

## Abstract

**Introduction:**

Post-Traumatic Stress Disorder (PTSD) in children is a complex condition that may go beyond the typical symptoms of re-experiencing, avoidance, and hyperarousal. Traumatic experiences can manifest through somatic or conversion symptoms, reflecting nonverbal expressions of distress. Repeated trauma exposure also increases the risk of comorbid anxiety disorders, such as panic disorder, highlighting the need for a comprehensive, trauma-informed approach.

**Objectives:**

Post-Traumatic Stress Disorder (PTSD) in children and adolescents can present with a wide range of symptoms, including somatic complaints, dissociation, and functional neurological symptoms. We report a clinical case that highlights the link between cumulative trauma and conversion disorder in a pediatric patient.

**Methods:**

M.N., an 11-year-old boy, was referred to child psychiatry after developing progressive tetraplegia over a two-month period, with no abnormalities found on medical investigations. His parents reported a history of physical assault by a classmate five months earlier, followed by loss of consciousness and initial trauma-related symptoms. Shortly afterward, the patient began experiencing falls with unresponsiveness, worsening to complete immobility. On admission, he was bedridden, emotionally labile, reported generalized pain and vertigo, and showed marked avoidance and re-experiencing symptoms suggestive of PTSD. The clinical picture also revealed features consistent with conversion disorder.

**Results:**

Following parental guidance and the introduction of SSRIs, the patient gradually recovered motor function and verbalized another traumatic experience: the murder of a neighbor, which had occurred a year earlier. He described vivid flashbacks involving visual, olfactory, and emotional triggers, and persistent fear of being killed in the same way. This second trauma had been previously unrecognized. His presentation combined both PTSD symptoms and functional neurological symptoms, reflecting a complex interaction between psychological trauma and somatic expression.

**Conclusions:**

This case underscores the importance of recognizing conversion disorder as a possible expression of PTSD in children, especially when symptoms are disproportionate or medically unexplained. It also highlights that multiple traumatic events, not a single one, often underlie such presentations. A comprehensive, trauma-informed approach is essential.

**Disclosure of Interest:**

None Declared

